# Building a pipeline of community-engaged researchers: How interdisciplinary translational research training programs can collaborate with their Community Research Advisory Councils

**DOI:** 10.1017/cts.2021.818

**Published:** 2021-07-14

**Authors:** Sarah E. LaFave, Duane J. Wallace, Raneitra Grover, Roger Clark, Stacey Marks, Cyd Lacanienta, Crystal Evans, Graziela Z. Kalil, Pamela Ouyang, Cheryl R. Himmelfarb, Martha Abshire

**Affiliations:** 1 Johns Hopkins University School of Nursing, Baltimore, MD, USA; 2 Morgan State University School of Community Health and Policy, Baltimore, MD, USA; 3 Johns Hopkins Institute for Clinical & Translational Research, Baltimore, MD, USA; 4 University of Maryland School of Medicine, Baltimore, MD, USA; 5 University of Maryland Baltimore Institute for Clinical & Translational Research, Baltimore, MD, USA; 6 Johns Hopkins University School of Medicine, Baltimore, MD, USA

**Keywords:** Community-engaged research, early career, training, advisory board, advisory council

## Abstract

Community research advisory councils (C-RAC) bring together community members with interest in research to support design, evaluation, and dissemination of research in the communities they represent. There are few ways for early career researchers, such as TL1 trainees, to develop skills in community-engaged research, and there are limited opportunities for C-RAC members to influence early career researchers. In our novel training collaboration, TL1 trainees presented their research projects to C-RAC members who provided feedback. We present on initial evidence of student learning and summarize lessons learned that TL1 programs and C-RACs can incorporate into future collaborations.

## Introduction and Rationale

Partnerships between researchers and community members can improve the relevance and impact of science.^1^ Community members can inform the development of research questions^2^ (i.e., identifying the problems that need solving), methods^2^ (i.e., how best to solve them), and dissemination^2^ (i.e., reaching people who could most benefit from study findings), and they can inform evaluation of research and funding proposals.^3,4^ These partnerships should be developed with intention: they will be most successful when both parties have the necessary training and resources.^5^ Strategies such as facilitating capacity-building workshops for community members and developing guidance on best practices for reseachers have been used successfully to strengthen community–researcher partnerships.^6,7^

Community Research Advisory Councils (C-RACs), also known as Community Advisory Boards, create a forum for patients, local residents, health professionals, and other stakeholders to ensure that research is beneficial and responsive to the priorities of the communities in which the research is conducted.^8,9^ The C-RAC at Johns Hopkins (JH), founded in 2009, provides substantive input to research teams on how to successfully and equitably engage with communities in research planning, conduct, and dissemination. The C-RAC includes 23 representatives from community organizations, academic and health systems partners, clinical research networks, health departments, advocacy groups, and the business sector. The C-RAC has a strong track record of collaborating with faculty researchers but, historically, has not advised or collaborated with pre-doctoral researchers.

The Johns Hopkins Institute for Clinical and Translational Research (ICTR) and University of Maryland Baltimore ICTR TL1 Programs partner to train students pursuing careers in clinical and translational research. The program provides funding and training for students from the Johns Hopkins Schools of Medicine, Nursing, and Public Health and Center for BioEngineering Innovation & Design, the Morgan State University Doctor of Public Health Program and PhD in Bioenvironmental Science Program, as well as both predoctoral and postdoctoral trainees from within the seven schools on the University of Maryland Baltimore Campus: Dentistry, Law, Medicine, Nursing, Pharmacy, Social Work, and the Graduate School.

The TL1 program is a one-to-two year-long experience that provides trainees with mentorship on their research projects and professional development opportunities such as training in abstract writing, developing a resume, and creating a scientific poster. The program aims to develop a diverse, interdisciplinary workforce of clinical and translational researchers who are prepared to engage with the community. However, in the past, the program has not provided formal training on community-engaged research (CEnR). Recognizing this gap, the program leadership sought to create a meaningful opportunity for the current cohort to not just learn about community engagement, but to leverage CEnR to improve the quality and relevance of their own research projects.

## Unmet Need and Purpose

C-RACs play an important role in helping research teams to develop, implement, and disseminate their studies, but there are few opportunities for C-RACs to engage with early career researchers. C-RAC members may be particularly interested in engaging with early career researchers because there is potential to not only contribute to the full trajectory of their studies from inception to dissemination but also to influence the researchers’ future interest in community collaboration. In turn, trainees may be eager for community insight on their studies, but be insufficiently equipped to engage community leaders. Little is known about training early career researchers in best practices for CEnR and many trainees may undergo little or no formal training on the topic.^10,11^

To intentionally expose our TL1 trainees to best practices in CEnR and to ensure that their research projects were informed by community members’ priorities and experiences, we developed a formal partnership with the JH C-RAC. The purpose of this article is to describe the collaboration between the C-RAC and TL1 programs as a model for training early career researchers in CEnR.

### Target Audience

Our findings may be of particular interest to faculty and administrators of clinical and translational research training programs, C-RAC leadership, and, more generally, to individuals who are interested in developing CEnR training for early career researchers.

## Methods

### Planning and C-RAC Preparation

Our TL1 team approached the C-RAC to explore the possibility of trainees presenting on their research to the C-RAC. Our TL1 program manager presented the idea during an in-person meeting with the C-RAC’s Community Relations Coordinator who then presented the idea to all C-RAC members. The C-RAC was enthusiastic to engage students and had limited previous opportunities to do so. The development and design of the partnership was strongly rooted in shared values for social justice and health equity, and our pedagogy was influenced by social critical theory.^12^ At the inception of our partnership, our TL1 and C-RAC program leadership teams met to discuss goals and a strategy for implementation. During those initial meetings, we determined that we should each provide orientation and clear expectations to our participants (C-RAC members and TL1 trainees). We also discussed logistical considerations, such as how the C-RAC could accommodate 15 trainee presentations and discussions into their existing meeting schedule. C-RAC leadership committed to devoting 5 full meetings to trainees and to reviewing trainee presentation slides prior to the meetings. The C-RAC leadership invited TL1 faculty to introduce the TL1 program to the C-RAC members to prepare members. During that introduction, faculty provided context on the purpose and format of the TL1 program, the career stage of trainees, and the purpose of the collaboration.

### TL1 Training

Faculty experts in CEnR developed and presented a training module to trainees at the beginning of their TL1 experience. The module was developed specifically for the TL1 trainees and provided an overview of the value of CEnR and best practices for incorporating community stakeholder input into the design, implementation, and dissemination of research. The TL1 program manager also provided an overview of the TL1-C-RAC collaboration. The TL1 team provided trainees with a presentation template as well as guidelines for presentation duration (10 minutes presentation followed by 10 minutes of discussion) and content. In individual meetings, faculty and staff mentors supported students in eliminating extraneous detail, using language and reading level appropriate for a lay audience, and highlighting the most important parts of the study on which the C-RAC would be best prepared to advise (e.g. recruitment strategies, overall study purpose and design, dissemination plans, equity and inclusion considerations). Each trainee met with the C-RAC staff to review and discuss suggested edits to their draft presentation. Trainees also attended a senior researcher’s presentation of her own research to the C-RAC during a regularly scheduled C-RAC research consultation; this observation experience provided a model for trainees on how to present to and facilitate feedback from the C-RAC.

### Plan for Trainee Presentations and C-RAC Feedback

Between August and December 2020, we planned that each of the 15 TL1 trainees would present their research to the C-RAC via video conference. During the discussion portion, C-RAC members would pose clarifying questions and provide insight on trainee-identified questions and concerns about their studies. Following trainee presentations and discussions, the C-RAC staff would provide written feedback via email to each trainee. This feedback would include a summary of the discussion, resources and contacts suggested by the C-RAC, and additional constructive critique that C-RAC members did not have time to provide during the meeting.

## Methods of Evaluation

The TL1 program administered two C-RAC-specific questions to trainees as part of their mid-year program evaluation. Trainees answered a five-point Likert-scale question, “To what extent do you agree with the following statement?: *Presenting my research to the Community Research Advisory Committee (C-RAC) enhanced my research experience.*” Students were also asked to provide feedback in a free-text field that prompted, “Briefly describe the following: 1. What did you think was valuable about the C-RAC experience; 2. How might we change the experience to improve it for future trainees?”

C-RAC debriefed the collaboration during two of its meetings. Members provided feedback on the presentations, meeting format, and their experiences engaging in the sessions in a group discussion format facilitated by the C-RAC Community Relations Coordinator. Program leaders from both the TL1 program and the C-RAC also maintained process notes to document their reflections on the pilot collaboration. Partners debriefed and reflected on the midyear presentations in December and met again in March to prepare for future collaboration.

## Results and Impact on Learning

Each of the 15 TL1 trainees successfully presented to the C-RAC on their research, and each trainee received verbal and written feedback on their work from the C-RAC. Trainees adhered to the ten-minute presentation guidelines. In some cases, they were not able to complete their full presentations because C-RAC members asked questions or engaged in discussion before the completion of the presentations.

**Fig. 1.**
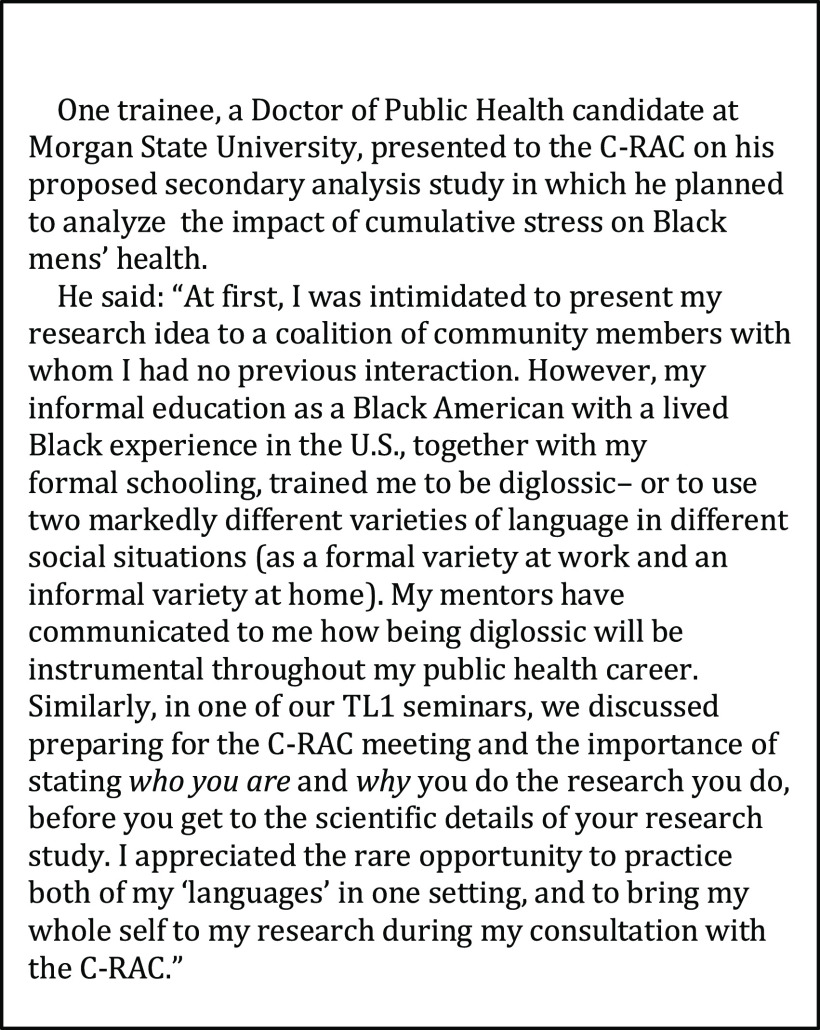
One trainee’s experience.

All 15 trainees completed the TL1 mid-year program evaluation. Twelve strongly agreed that presenting to C-RAC enhanced their research experience, three were neutral, and none disagreed or strongly disagreed. In their qualitative responses, trainees commented that benefits included gaining insight on methods from community stakeholders, practicing presenting on their work to a lay audience, hearing community members’ recruitment ideas (and, in some cases, specific contacts), and reflecting on the potential impact of their research. One trainee wrote, “Presenting to C-RAC made me stop to reflect about the ultimate goal of my research and how it can translate to a positive impact on the greater community. It allowed me to think about how to present information effectively.” In Fig. 1, we provide more detail about one trainee’s experience.

Trainees also provided constructive feedback including 1) the presentation time should be lengthened to allow trainees to provide context for their studies, 2) program leadership should provide a sample presentation, and 3) program leadership should clarify expectations for next steps following the presentations. One trainee suggested, “It would be helpful for trainees to see examples of previous presentations and a bit more direction of what is expected in the C-RAC presentation beforehand.”

Program leaders noted that it would be helpful in the future for trainees to more thoroughly describe the stage of their study and the type of data available. Some of the trainees are doctoral candidates pursuing dissertation research, which frequently involves secondary analysis. Because those details were not provided to the C-RAC, members sometimes offered feedback that could not be implemented due to the stage or type of study.

## Future Plans and Recommendations

Trainees and C-RAC members were overwhelmingly positive about this pilot collaboration. In their debrief discussions, C-RAC members expressed that trainee presentations had generally been appropriate in length and content, but felt that trainees should further emphasize the potential impact of their projects and that they should provide more clarification about their limitations (e.g., inability to add variables if using secondary data). They expressed strong interest in re-engaging with the trainees during the year. Our team suggested a small group room format for the second round of presentations, to limit the time burden on C-RAC members. However, C-RAC members adamantly supported a format that would allow all C-RAC members to hear full-length follow-up presentations by all trainees. The C-RAC members extended an invitation to TL1 trainees for 15-minute follow-up presentations at the end of the TL1 training year. They requested that trainees provide an update on their projects and how they incorporated C-RAC feedback into their studies and to clearly state the direct and indirect benefits to the community in these final presentations.

**Fig. 2.**
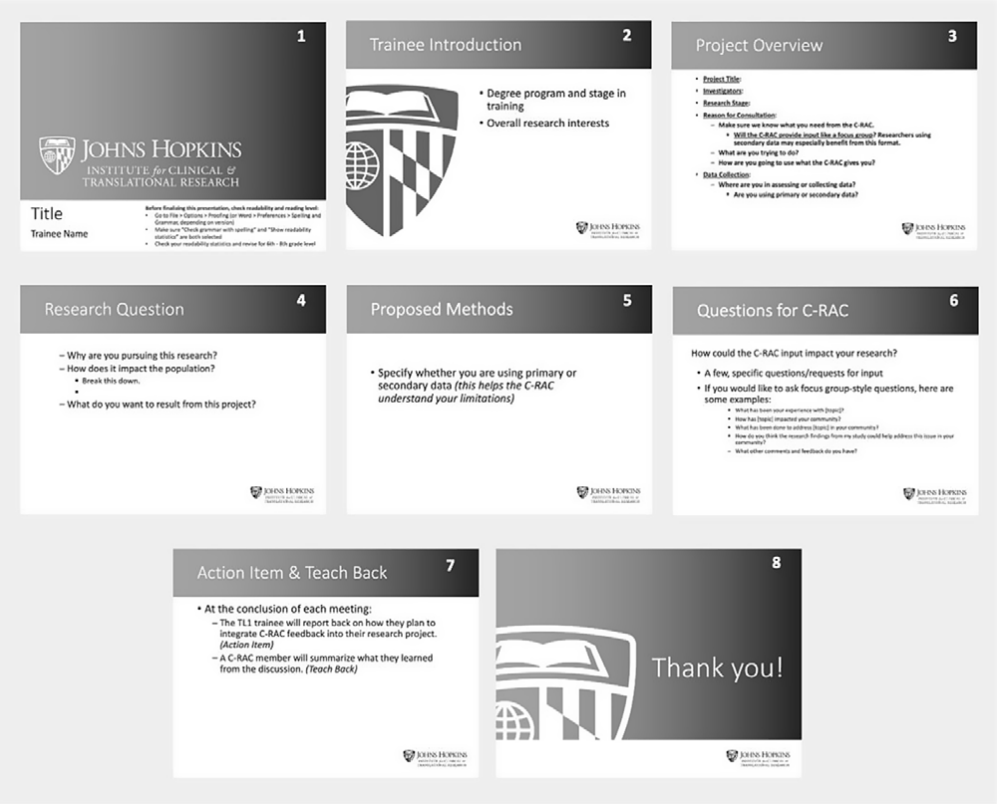
Trainee presentation template.

We recommend a similar partnership for other clinical and translational research training programs. Program leaders should partner with their C-RAC (or other community stakeholder group) from the inception of the collaboration to ensure that it meets both programs’ goals and fits within both programs’ logistical constraints. Faculty and staff should provide explicit expectations for trainees, including a presentation template as well as sample presentation, guidelines for duration and content of presentations, and guidelines for incorporating feedback into studies and other next steps following the presentations. We iteratively refined our guidelines for trainees throughout this process and provide our revised slide template in Fig. 2.

It is also important to prepare the community stakeholder group by providing information on the trainees’ stage of research and how community input can impact research at those stages. In the future, our C-RAC leadership plans to provide a brief introduction to study designs and methods, including secondary data analysis, and to provide examples of the type of input that would be beneficial to trainees.

Intentionally exposing early career researchers to supportive, structured opportunities for community engagement fosters community buy-in and bidirectional learning throughout the research process and has the potential to develop a pipeline of CEnRs. Ultimately, these partnerships could contribute to more relevant and impactful research and improved public health.
